# Alterations in Mitochondrial Morphology and Quality Control in Primary Mouse Lung Microvascular Endothelial Cells and Human Dermal Fibroblasts under Hyperglycemic Conditions

**DOI:** 10.3390/ijms241512485

**Published:** 2023-08-06

**Authors:** Natalia V. Belosludtseva, Dmitriy A. Serov, Vlada S. Starinets, Nikita V. Penkov, Konstantin N. Belosludtsev

**Affiliations:** 1 Institute of Theoretical and Experimental Biophysics, Russian Academy of Sciences, Institutskaya 3, 142290 Pushchino, Russia; vlastar@list.ru; 2 Prokhorov General Physics Institute of the Russian Academy of Sciences, Vavilov St. 38, 119991 Moscow, Russia; dmitriy_serov_91@mail.ru; 3 Institute of Cell Biophysics, Russian Academy of Sciences, Institutskaya 3, 142290 Pushchino, Russia; nvpenkov@rambler.ru; 4 Department of Biochemistry, Cell Biology and Microbiology, Mari State University, pl. Lenina 1, 424001 Yoshkar-Ola, Russia

**Keywords:** diabetic hyperglycemia, mitochondrial dysfunction, mitochondrial morphology, mitochondrial dynamics, mitophagy, mitochondrial biogenesis

## Abstract

The effect of hyperglycemia on the morphology of individual mitochondria and the state of the mitochondrial network in primary mouse lung microvascular endotheliocytes and human dermal fibroblasts has been investigated. The cells were exposed to high (30 mM) and low (5.5 mM) glucose concentrations for 36 h. In primary endotheliocytes, hyperglycemic stress induced a significant increase in the number of mitochondria and a decrease in the interconnectivity value of the mitochondrial network, which was associated with a decrease in the mean size of the mitochondria. Analysis of the mRNA level of the genes of proteins responsible for mitochondrial biogenesis and mitophagy revealed an increase in the expression level of the *Ppargc1a*, *Pink1*, and *Parkin* genes, indicating stimulated mitochondrial turnover in endotheliocytes under high glucose conditions. In primary fibroblasts, hyperglycemia caused a decrease in the number of mitochondria and an increase in their size. As a result, the mitochondria exhibited higher values for elongation. In parallel, the mRNA level of the *Ppargc1a* and *Mfn2* genes in fibroblasts exposed to hyperglycemia was reduced. These findings indicate that high glucose concentrations induced cell-specific morphological rearrangements of individual mitochondria and the mitochondrial network, which may be relevant during mitochondria-targeted drug testing and therapy for hyperglycemic and diabetic conditions.

## 1. Introduction

Diabetes mellitus is one of the most common diseases worldwide, associated either with impairment of insulin secretion (type 1 diabetes) or with the tolerance of the body’s cells to the action of the hormone (type 2 diabetes). In both cases, a common pathological change is a chronic increase in blood glucose level—diabetic hyperglycemia, which is causally linked to vascular complications and further extensive damage to many organs and tissues [1].

One of the key processes underlying the pathogenesis of diabetes and hyperglycemic damage to cells is the development of mitochondrial dysfunction and associated oxidative stress [2,3]. Recent studies have shown that mitochondria-targeted drugs suppress the main pathological manifestations and normalize energy metabolism and redox balance in both diabetic animals and patients [3,4,5].

Despite the generally recognized role of mitochondrial dysfunction in the pathogenetic mechanisms of diabetes and hyperglycemia, cell-specific features of organellar responses to these conditions are not well understood. It was found that mitochondrial oxidative phosphorylation, the main function of the organelles and a critical ATP-generating metabolic pathway, was suppressed in the most energy-consuming tissues of diabetic animals, whereas it was not altered in other animal tissues [2,6]. Some studies revealed that in animal models of type 2 diabetes, mitochondria in skeletal and cardiac muscle cells became more susceptible to the calcium-dependent permeability transition pore (PTP) opening, while liver mitochondria demonstrated increased resistance to pore formation [7,8,9]. Several lines of evidence indicated that diabetes-induced changes in the expression of a substantial proportion of mitochondrial membrane ion channels and exchangers occurred in opposite directions in cells of different tissues [10,11,12,13].

It is known that structural alterations both at the level of individual mitochondria and the mitochondrial network can modulate and coordinate the functions of mitochondria to support cells facing a variety of functional demands and metabolic disorders [14]. The shape of individual mitochondria determines their surface area available for local interactions with other organelles relative to the volume of the mitochondrial matrix. The dynamic behavior of the mitochondrial network ensures the distribution and adequate functioning of the organelles in cells, allows their adaptation to pathophysiologic states, and contributes to quality control, including the processes of mitochondrial fusion/fission, biogenesis, and mitophagy [2]. Previous studies demonstrated that mitochondrial quality control is causally related to hyperglycemia and metabolic diseases, but the specific changes in these mechanisms may differ depending on the cell type [2,5]. In particular, the transcriptional activator PGC-1α, a key regulator of mitochondrial biogenesis, was significantly upregulated in diabetic hepatocytes but not in most other cells and tissues of animals with diabetes [15,16,17].

Therefore, a cell-specific analysis of changes in the structure and function of mitochondria in hyperglycemic stress and diabetes is further required. As is known, cells of the vascular system, including endotheliocytes and fibroblasts, are at the forefront and most vulnerable to the effects of prolonged elevated plasma glucose levels, and their damage can lead to the development of diabetic micro- and macrovascular complications [18,19]. This study intended to analyze structural and dynamic changes in mitochondria in primary endotheliocytes and fibroblasts exposed to high glucose concentrations (30 mM glucose for 36 h). We have evaluated the effects of hyperglycemic stress on the membrane potential of mitochondria and their morphology using four parameters: number, size (perimeter), elongation, and interconnectivity. We have also compared these effects with the mRNA expression level of proteins responsible for mitochondrial biogenesis, mitochondrial dynamics, and mitophagy in the cell.

## 2. Results

### 2.1. Hyperglycemia Induces a Decrease in the Membrane Potential of Mitochondria in Primary Endotheliocytes and Fibroblasts

**Figure 1:**
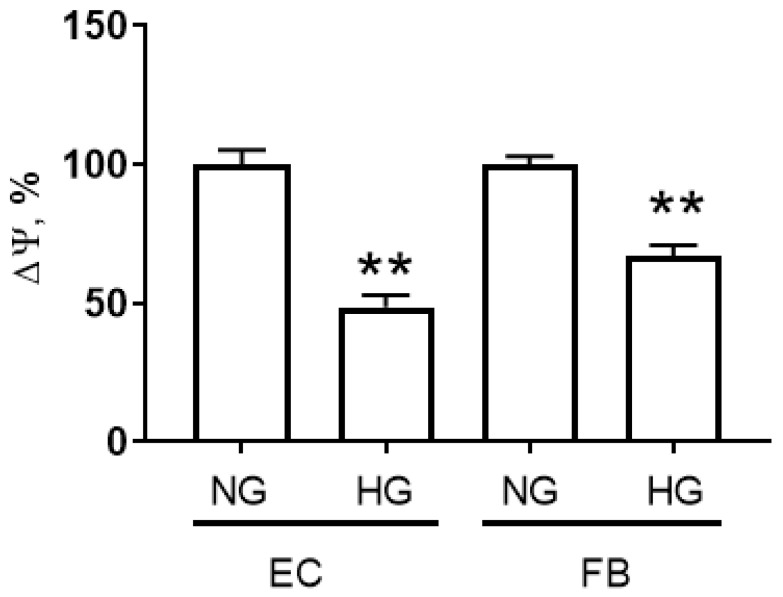
Changes in mitochondrial membrane potential (ΔΨ) in primary mouse lung microvascular endotheliocytes (EC) and human dermal fibroblasts (FB) exposed to hyperglycemic conditions (30 mM D-glucose) for 36 h. Data represent the mean ± standard error of the mean of 5–6 biological replicates. **—differences between normoglycemia (NG, 5.5 mM D-glucose) and hyperglycemia (HG, 30 mM D-glucose) are significant, *p* < 0.01.

The depolarization of the inner mitochondrial membrane is one of the typical manifestations of mitochondrial damage in hyperglycemia. Here, we evaluated the effect of 5.5 mM and 30 mM of glucose on the membrane potential of mitochondria in the primary cultures of mouse lung microvascular endotheliocytes and human dermal fibroblasts after 36 h of culture. As can be seen from Figure 1, the high concentration of glucose caused a drop in the mitochondrial membrane potential in both types of cells.

These results indicate that primary endotheliocytes and fibroblasts exposed to hyperglycemic stress displayed mitochondrial dysfunction.

### 2.2. Hyperglycemia Promotes Bidirectional Changes in the Morphology of Individual Mitochondria in Primary Mouse Lung Microvascular Endothelial Cells and Human Dermal Fibroblasts

Alterations in mitochondrial mass and morphology are associated with the progression of diabetes and hyperglycemic damage to cells. Therefore, we next analyzed the effect of hyperglycemia on the number and morphological features of the organelles in primary endothelial cells and fibroblasts. In order to quantify the changes in mitochondrial morphology, at least 20 images of each cell type were analyzed. The values of the number of mitochondria per cell, mean perimeter, interconnectivity of the mitochondrial network, and elongation of individual mitochondria were calculated as indicators of their normal distribution and morphology.

**Figure 2:**
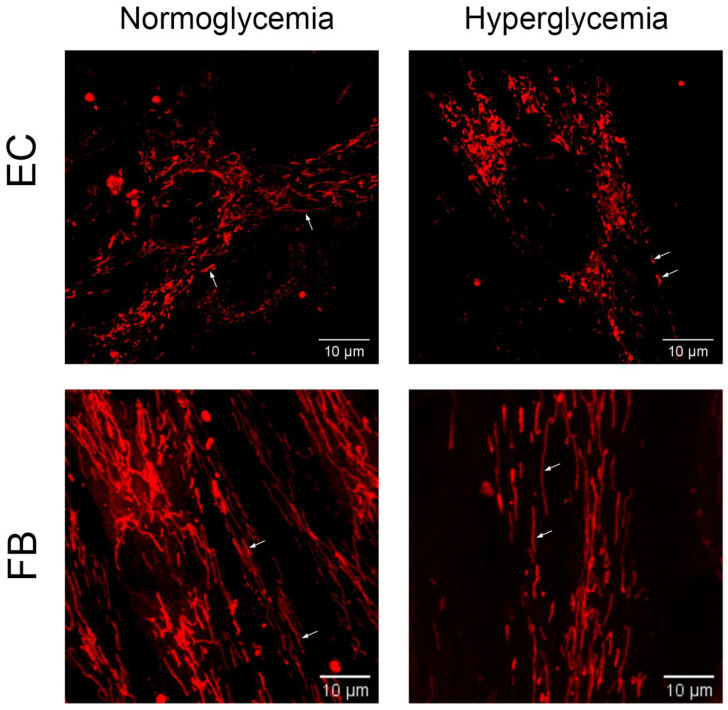
Representative images showing the morphology of MitoTracker Red-labeled mitochondria in primary human dermal fibroblasts (FB) and mouse lung microvascular endothelial cells (EC) cultured under control conditions (normoglycemia, 5.5 mM D-glucose) and 36 h hyperglycemia (30 mM D-glucose). Mitochondria of control endotheliocytes (upper left panel) are larger and more often associated with each other (white arrows). In endotheliocytes exposed to high glucose concentrations (upper right panel), mitochondria showed a decrease in their size, and fragmentation of the mitochondrial network was observed (white arrows). An increase in the size and elongation of individual mitochondria was shown in fibroblast cell cultures after exposure to hyperglycemia (bottom right panel, white arrows) compared to the control (bottom left panel, white arrows).

Figure 2 and Table 1 show that in endotheliocytes, hyperglycemia caused an increase in the number of mitochondria by 1.75 times, a reduction in their perimeter by 1.12 times, and a decrease in the interconnectivity value by 1.13 times, suggesting the fragmentation of the mitochondrial network. Conversely, in fibroblasts, hyperglycemia led to a decrease in the number of mitochondria by 1.3 times and an increase in their perimeter by 1.14 times. Individual mitochondria in fibroblasts exposed to high glucose conditions demonstrated higher values of elongation, and the interconnectivity value of the mitochondrial network was unaffected.

### 2.3. Effect of Hyperglycemia on the mRNA Expression of Proteins Responsible for Mitochondrial Biogenesis, Mitochondrial Dynamics, and Mitophagy in Primary Endotheliocyte and Fibroblast Cultures

**Figure 3:**
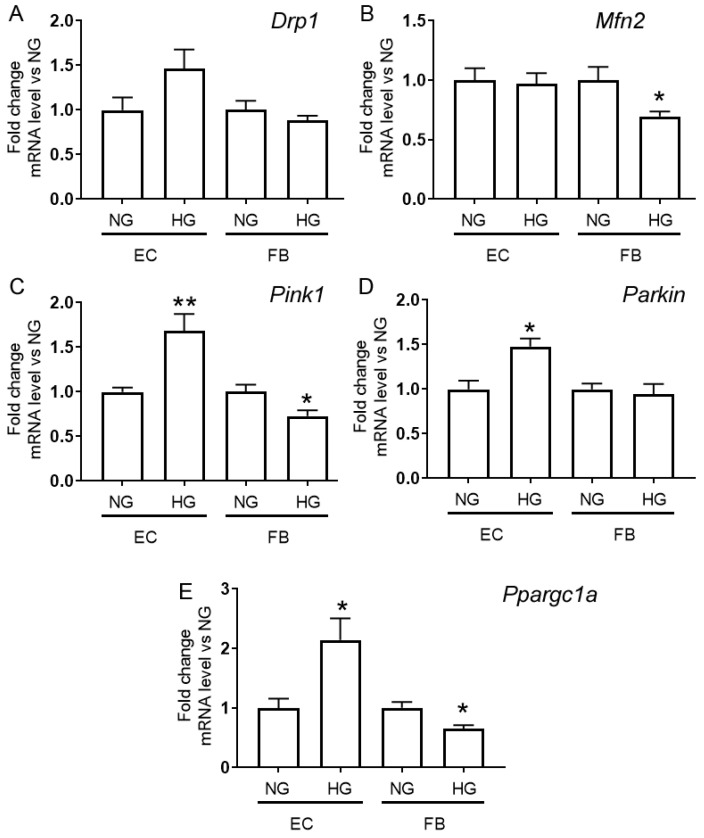
Effect of hyperglycemia treatment on mitochondrial quality control (the processes of mitochondrial fusion/fission, biogenesis, and mitophagy) in primary endotheliocytes and fibroblasts. The relative mRNA levels of the *Drp1* (**A**), *Mfn2* (**B**), *Pink1* (**C**), and *Parkin* (**D**) *Pparpgc1a* (**E**) genes in primary mouse lung endothelial cells (EC) and human dermal fibroblasts (FB) after 36 h incubation with 5.5 mM D-glucose (NG) or 30 mM D-glucose (HG). Data represent the mean ± standard error of the mean of 5 biological replicates. *—differences between normo- (NG) and hyperglycemia (HG) are significant, *p* < 0.05. **—differences between NG and HG are significant, *p* < 0.01.

To determine if the morphologic changes in mouse lung microvascular endotheliocyte and human dermal fibroblast mitochondria under high glucose conditions are associated with the mitochondrial quality control system, we evaluated the level of expression of genes encoding proteins responsible for mitochondrial biogenesis, fission/fusion, and mitophagy in the experimental groups (Figure 3).

Figure 3 shows that endothelial cells preconditioned in high glucose conditions displayed an increased level of mRNA of *Ppargc1a* encoding the PGC-1α protein responsible for mitochondrial biogenesis and elevated mRNA levels of the *Pink1* and *Parkin* genes encoding proteins responsible for mitophagy. An increase in the mRNA content of these genes may indirectly indicate the stimulation of mitochondrial biogenesis and mitophagy and, hence, a more active turnover of mitochondria in endotheliocytes exposed to hyperglycemia. In addition, an elevated level of the gene expression of the transcriptional coactivator PGC-1α, one of the key regulators of biogenesis, may be responsible for the increase in the number of mitochondria in endothelial cells in hypoglycemia.

In human skin fibroblasts exposed to high levels of glucose, the mRNA content of the *Ppargc1a* gene was reduced, which could be the cause for the decrease in the number of mitochondria in these cells. In parallel, a decline in the mRNA level of the Mfn2 gene encoding mitofusin-2, which is responsible for mitochondrial fusion, was observed.

## 3. Discussion

Increasing evidence points to mitochondrial dysfunction as a common pathogenic factor in hyperglycemia and diabetes-related vascular complications in both human pathology and cellular and animal models [2,3,4,20]. Mitochondria can sense hyperglycemic stress and support cell survival or death through differential modulation of mitochondrial morphology, fission/fusion, biogenesis, mitophagy, and essential functions, thereby providing adaptive and maladaptive cellular stress responses [2,21]. Our findings showed that in primary mouse lung microvascular endothelial cells and human dermal fibroblasts exposed to 30 mM glucose for 36 h, multidirectional changes were observed in the number of mitochondria, the morphology of individual mitochondria, and the state of the mitochondrial network, which led to the development of mitochondrial depolarization in these cells. Incubation of primary endotheliocytes with high glucose concentrations increased the number of mitochondria, decreased the perimeter of the organelles, and stimulated the fragmentation of the mitochondrial network. These events were accompanied by an increase in the expression level of the *Ppargc1a* gene, which encodes PGC-1α, a protein that upregulates the transcription of genes involved in mitochondrial biogenesis. In parallel, there was an increase in mRNA levels of *Pink1* and *Parkin*, which are linked to the clearance of damaged mitochondria via mitophagy to perform the mitochondrial quality control function. In primary fibroblasts, high glucose concentrations reduced the number of mitochondria and induced their elongation, which resulted in an increase in the mean perimeter of mitochondria. These changes were accompanied by a decrease in the mRNA levels of *Ppargc1a* and *Mfn2*, which encodes mitofusin-2, a protein that is required for mitochondrial fusion. Taken together, our results suggest that the cellular and mitochondrial response to the development of hyperglycemic stress is tissue- and cell-specific.

Reshaping of mitochondria and remodeling of the mitochondrial network control different cellular functions, such as the maintenance of the membrane potential, ATP synthesis, ROS generation, respiration, ion transport, signaling pathways, and adaptation to stressful conditions [22,23,24]. The synthesis of ATP is known to use the mitochondrial membrane potential, which is a global indicator of mitochondrial function [23]. As mentioned earlier, there is evidence that the mitochondria in cells of different organs may exhibit different functional responses to an excess of substrates and may vary in their sensitivity to metabolic pathologies [2,6]. Moreover, it is known that cells from different sources may vary substantially in terms of their mitochondrial mass, function, and morphology, which may, at least in part, underlie differences in their susceptibility to elevated glucose levels [2,14,24]. Our results indicate that cell-specific morphological rearrangements of individual mitochondria and the mitochondrial network may be responsible for the heterogeneity of the functional activity of mitochondria in vascular cells under high glucose conditions.

The structural and dynamic changes in mitochondria ensure cell adaptation to the conditions of excessive levels of metabolic substrates by regulating mitochondrial distribution and function, including the membrane potential value, and also contribute to the quality control of the organelles [21,24]. Our results indicate that endothelial cells exhibit more plastic states and respond with adaptive changes aimed at the stimulation of mitochondrial turnover by increasing biogenesis and clearance of fragmented mitochondria in response to hyperglycemia. On the contrary, primary fibroblasts exposed to hyperglycemia show decreased mitochondrial mass and progressive elongation of individual mitochondria in association with decreased mitochondrial fusion, which hinders mitophagy and may be associated with increased ROS production and mitochondrial DNA damage [25]. Some studies have also revealed differentiated mitochondrial function in fibroblasts and endothelial cells in response to chemical exposure, with fibroblasts showing significantly higher relative sensitivity to many agents with toxic potential [26]. 

Mitochondria play a key role in hyperglycemia-induced metabolic reprogramming and oxidative damage, being both the main site of ROS formation and targets for their attack [3]. Some studies have shown that remodeling the mitochondrial network can reduce the overproduction of ROS and promote the integrity of mitochondrial DNA [27,28]. In addition, a decrease in the mitochondrial membrane potential by several percent, which may be observed during mitochondrial fragmentation, can lead to a significant decrease in the formation of ROS in the mitochondrial respiratory chain [23,29]. In this regard, targeting mitochondrial network remodeling upon diabetic hyperglycemia may be an important therapeutic strategy, and exposure to several mitochondrial quality control mechanisms may have broader effectiveness for activating adaptive responses. Moreover, the mitochondria-directed treatment strategies for cells of different types should be adjusted depending on the specific response of the cell to hyperglycemic conditions.

It should be noted that the current study has some limitations. In our study, we used two types of cells obtained from two different tissues and organisms, namely endothelial cells derived from microvessels of mouse lungs and fibroblasts of human skin. Despite the fact that a drop in the membrane potential of mitochondria was observed in both types of cells, the species origin of the cells may also affect changes in the morphology of mitochondria in hyperglycemia. Moreover, differences in the response of the mitochondrial reticulum could be induced differently in the different cell types of the various anatomical districts. Therefore, there could be different behaviors between fibroblasts of different anatomical districts or different types of endotheliocytes. Further research is needed to identify the species- and tissue-specific cellular response to the development of diabetic hyperglycemia.

## 4. Materials and Methods

### 4.1. Isolation and Culture of Primary Mouse Lung Microvascular Endothelial Cells and Human Dermal Fibroblasts

Isolation of endothelial cells from the microvessels of mouse lungs (BALB/c, males weighing 20–22 g) was performed by the method of magnetic separation using antibodies against CD31 [30,31]. The purity of the culture was examined by staining with primary anti-CD31 and AF488-conjugated secondary antibodies. All the protocols were carried out in accordance with EU Directive 2010/63/EU for animal experiments and approved by the Ethics Committee at the Institute of Theoretical and Experimental Biophysics, Russian Academy of Sciences (Protocol No. 19/2023 of 18.02.2023). The primary culture of human skin fibroblasts was kindly provided by the staff of the Institute of Regenerative Medicine of Lomonosov Moscow State University (Biobank collection No.: MSU_FB (Available online: https://human.depo.msu.ru (accessed on 5 August 2023). Cells were cultured according to the standard protocols [30]. All experiments were carried out using cells that were matched for passages 7–13 across experimental groups. Survival cells were monitored by propidium iodide staining. Samples with a survival rate of at least 95% were used in the experiments. 

Before the experiments, the cells were grown on 25 mm circular cover glasses (Heinz Herenz, Hamburg, Germany) pretreated with 0.2% gelatin (PanEco, Moscow, Russia) and placed in 6-well plates. Cells were cultured on glasses in the basal media containing 5.5 mM D-glucose for 3 days until a confluence of 90% was achieved. The basal medium with 5.5 mM D-glucose for normal conditions was supplemented with 24.5 mM D-glucose to bring the concentration to 30 mM in the high glucose media. Hyperglycemic stress was induced by culturing cells in the medium with 30 mM glucose for 36 h. Control cells were incubated for 36 h with the basal medium containing 5.5 mM D-glucose. For each of the experimental measurements, the biological replicates (N) refer to cells that were cultured, passaged, and treated separately.

### 4.2. Mitochondrial Membrane Potential Assay

To assess the membrane potential of the inner mitochondrial membrane, the fluorescent dye rhodamine 123 (ThermoFisher, Waltham, MA, USA) was used [30]. The cells were washed three times with Hanks’ balanced salt solution (HBSS) with an appropriate glucose concentration and stained with rhodamine 123 at a concentration of 2.5 µg/mL for 30 min at 37 °C. After the end of incubation, the cells were washed three times with HBSS (in the case of hyperglycemia, Hanks’ solution containing 30 mM glucose was used). Fluorescence intensity was recorded from individual cells in the field of view at the excitation and emission wavelengths of 488 and 528 nm, respectively, using the imaging system based on microscope Motic-AE31E (Motic, Barcelona, Spain) with objective Motic PLAN FLUAR 10×, N.A. 0.3, LEDs M340D3, M375D2 (Thorlabs Inc., Newton, NJ, USA), exposition time was 500 ms. To assess the value of the mitochondrial membrane potential, 2 µM FCCP was added (Sigma-Aldrich, Saint Louis, MO, USA). The resulting fluorescence intensity of rhodamine 123 was expressed as the ratio of the fluorescence intensity at the final time point (F) to the average fluorescence intensity under baseline conditions (F0). For the convenience of comparing data between two cell types, the mitochondrial membrane potential was expressed as a percentage. A change in the fluorescence intensity in cells under low glucose conditions was taken as 100%.

### 4.3. Assessment of the Morphology of Individual Mitochondria by Confocal Microscopy

**Figure 4:**
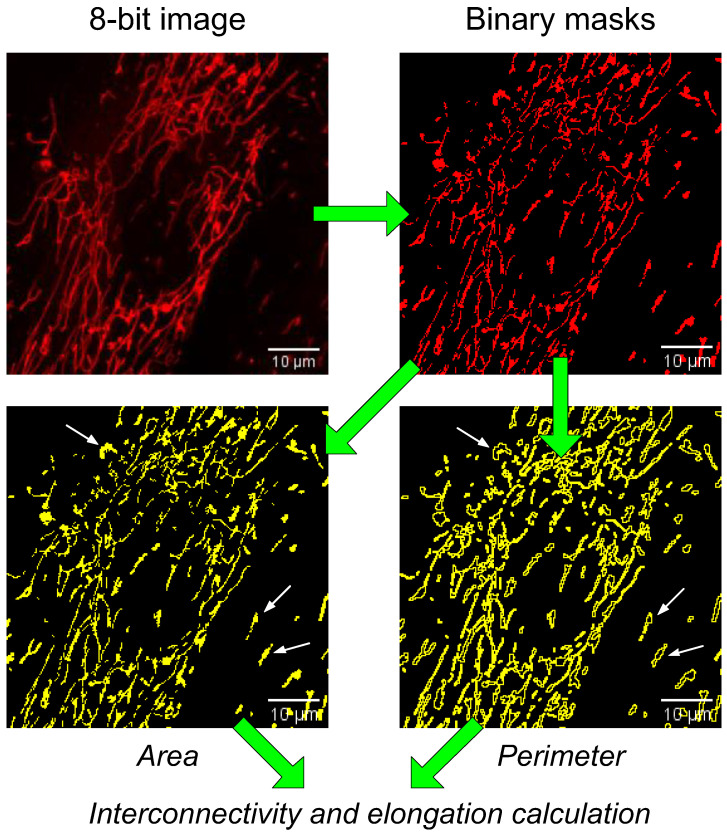
The algorithm for calculating the morphological characteristics of mitochondria. Formulas for calculating the values of interconnectivity and elongation are given below. Examples of graphical representations of the perimeter and area of mitochondria are shown with white arrows. The green arrows show the sequence of processing the images obtained.

Fluorescent staining of cells to determine the morphology of individual mitochondria was performed using the MitoTracker DeepRed probe (Thermo Fisher Scientific, Waltham, MA, USA). The cells were washed twice from the culture medium with HBSS (37 °C) and incubated with 200 nM MitoTracker DeepRed for 30 min at 37 °C. After incubation, the cells were washed with Hanks’ solution. Hanks’ solution with 30 mM glucose was used for cells in hyperglycemia. Confocal images were obtained using a DMI6000 microscope (Leica, Wetzlar, Germany). The images were further processed with the Image J2 (Fiji) software with Bio-Formats plugins (National Institutes of Health, Bethesda, MD, USA). The following parameters were calculated: the number of mitochondria per one cell, the mean perimeter of one mitochondrion, the value of interconnectivity, and the value of elongation, which were calculated using Formulas (1) and (2), respectively [32]. The algorithm for calculating the morphological characteristics of mitochondria is shown in Figure 4.

(1) Interconnectivity=mean area/mean perimeter

(2) $$\mathrm{Elongation}=\frac{1}{4*\pi *\left(\mathrm{mean}\;\mathrm{area}/{\mathrm{mean}\;\mathrm{perimeter}}^{2}\right)}$$

The interconnectivity value signifies the association of the mitochondrial network. Higher values for interconnectivity mean that mitochondria are more connected to the mitochondrial network and have more physical connections, while lower scores mean that mitochondria are more represented as individual organelles. The value of elongation makes it possible to evaluate the shape of individual mitochondria, and it is inversely proportional to circularity. Higher values for elongation signify more abstract shapes, while a value of 1 would be considered a circle. 

For each experimental condition, four biological replicates were performed. For each sample, at least five visual fields were analyzed.

### 4.4. RNA Extraction, Reverse Transcription, and Quantitative Real-Time PCR

The level of gene expression of proteins responsible for mitophagy, mitochondrial biogenesis, and mitochondrial dynamics was determined by real-time PCR with a reverse transcription step [30,33]. Total RNA from cell suspension samples was obtained using ExtractRNA reagent (Eurogen, Russia) according to the manufacturer’s protocol. Real-time PCR was performed using the QuantStudio 1 amplifier (Thermo Fisher Scientific, Waltham, MA, USA) using the qPCRmix-HS SYBR kit (Eurogen, Russia). The analysis of gene-specific primers was performed using Primer-BLAST. The lists of gene-specific primers are shown in Table 2. The calculation of the ∆∆Ct was performed according to the formula ∆∆Ct = ∆Ct (Control) − ∆Ct (Experiment); each value of ∆Ct was calculated with the formula ∆Ct = Ct (Gene of interest) − Ct (Rplp2) [33].

### 4.5. Statistical Analysis

The data were analyzed using GraphPad Prism 8.0 and were presented as mean ± standard error of the mean of 3–6 independent experiments. Statistical differences between the means were determined by the Mann–Whitney U test; *p* < 0.05 was considered to be statistically significant.

## 5. Conclusions

The present study demonstrated the oppositely directed changes in the mitochondrial count, the morphology of individual mitochondria, and the state of the mitochondrial network in primary mouse lung microvascular endothelial cells and human dermal fibroblasts in response to hyperglycemic exposure. The results are in line with the data that cells from different sources may vary substantially in terms of mitochondrial morphology and function in hyperglycemia, which may, at least partially, underlie differences in their susceptibility to elevated glucose levels; within one case, there may be an adaptive response to hyperglycemic stress, and in the other case—a maladaptive one. Differences in the response of the mitochondrial reticulum from blood vessel cells may be relevant during mitochondria-targeted therapy for hyperglycemic and diabetic conditions. Paying more attention to the specificity of such a response may also be helpful in interpreting data obtained from testing mitochondria-targeted agents.

## Figures and Tables

**Table 1:** Morphological analysis of mitochondria in mouse lung microvascular endothelial cells and human dermal fibroblasts exposed to normoglycemia (NG, 5.5 mM D-glucose) and hyperglycemia (HG, 30 mM D-glucose) for 36 h.

Parameters of Mitochondrial Morphology	Endotheliocytes		Fibroblasts	
	NG	HG	NG	HG
Number, pcs	359 ± 69	631 ± 122 *	792 ± 166	586 ± 141 *
Mean Perimeter, µm	3.7 ± 0.2	3.3 ± 0.2 **	5.46 ± 0.81	6.24 ± 0.86 *
Interconnectivity, a. u.	0.17 ± 0.01	0.15 ± 0.01 *	0.21 ± 0.01	0.21 ± 0.01
Elongation, a. u.	1.73 ± 0.02	1.74 ± 0.01	1.80 ± 0.10	1.95 ± 0.13 *

Values are given as mean ± standard error of the mean of 3–4 biological replicates. *—differences between normo- (NG) and hyperglycemia (HG) are significant, *p* < 0.05. **—differences between NG and HG are significant, *p* < 0.01.

**Table 2:** List of gene-specific primers for RT-PCR analysis.

Gene	Forward (5′→3′)	Reverse (5′→3′)
Gene-specific primers—Human		
*Pink1*	AGCCACCATGCCTACATTGC	TGGAGGAACCTGCCGAGATG
*Prkn*	ACAGCAGGAAGGACTCACCA	TGCTGCACTGTACCCTGAGT
*Drp1*	CTTCGGAGCTATGCGGTGGT	GCAGGACGAGGACCAGTAGC
*Mfn2*	AAGTGGAGAGGCAGGTGTCG	TCCTCTATGTGGCGGTGCAG
*Ppargc1a*	GCCTTCCAACTCCCTCATGG	CTCCGGAAGAAACCCTTGCAT
*Rplp2*	GACGACCGGCTCAACAAGGT	CCAATACCCTGGGCAATGACG
Gene-specific primers—Mouse		
*Pink1*	TTGCCCCACACCCTAACATC	GCAGGGTACAGGGGTAGTTCT
*Prkn*	AGCCAGAGGTCCAGCAGTTA	GAGGGTTGCTTGTTTGCAGG
*D* *rp* *1*	TTACAGCACACAGGAATTGT	TTGTCACGGGCAACCTTTTA
*Mfn2*	CACGCTGATGCAGACGGAGAA	ATCCCAGCGGTTGTTCAGG
*Ppargc1a*	CTGCCATTGTTAAGACCGAG	GTGTGAGGAGGGTCATCGTT
*Rplp2*	CGGCTCAACAAGGTCATCAGTGA	AGCAGAAACAGCCACAGCCCCAC

## Data Availability

The data presented in this study are available upon request from the corresponding author.
